# Targeted deletion of macrophage ferritin heavy chain protects from macrophage ferroptosis in acute respiratory distress syndrome

**DOI:** 10.21203/rs.3.rs-5276478/v1

**Published:** 2026-01-08

**Authors:** Suzanne Cloonan, William Zhang, Kihwan Kim, Lynne Faherty, Will Simmons, Sebastian Carrasco, Katherine Hoffman, Sean Houghton, Chia-Lang Hsu, Leora Haber, Parag Goyal, Kuei-Pin Chung, Karla Ballman, David Redmond, Joseph Mancias, Augustine Choi, Edward Schenck, Maria Plataki, Christopher Mason, Cem Meydan

**Affiliations:** Trinity College, Dublin; Weill Cornell Medicine; Weill Cornell Medicine; Trinity College Dublin, Ireland; Weill Cornell Medicine; MSKCC; Weill Cornell Medicine; Weill Cornell Medicine; Weill Cornell Medicine; Weill Cornell Medicine; College of Medicine, National Taiwan University; Mayo Clinic; Weill Cornell Medicine; Dana-Farber Cancer Institute; Weill Cornell Medicine; Weill Cornell Medicine; Weill Cornell Medicine; Weill Cornell Medicine; Weill Cornell Medicine

## Abstract

Ferritin, consisting of ferritin heavy chain (FTH1) and light chain (FTL) subunits, is an essential intracellular iron storage protein fundamental for cellular function. However, the source and the biological role of extracellular ferritin (ex-ferritin) are less understood. Recent studies have linked elevated serum ex-ferritin with adverse outcomes in individuals with acute respiratory distress syndrome (ARDS). In this study, we demonstrate that both FTH1 and FTL are significantly enriched in the serum, blood monocytes, and alveolar macrophages (AMs) of individuals with ARDS, a phenomenon we successfully replicate in a murine hyperoxia-induced acute lung injury (HALI) model. We show that FTH1 is consistently upregulated in macrophages during lung injury development, and mice with a targeted deletion of FTH1 in myeloid (LysMcre) or resident lung macrophage (Cd11ccre) populations exhibit attenuated HALI. This reduced injury is linked to macrophage resistance to ferroptotic cell death, ferritinophagy, altered airway inflammatory responses, and lower lung extracellular iron and higher levels of FTL-ex-ferritin. Transplantation of FTL-ex-ferritin-enriched bronchoalveolar lavage fluid to wild-type mice protected against HALI. The ratio of FTL-ex-ferritin to FTH1 in the serum of individuals with ARDS who died was higher than that of those that survived, suggesting that the balance between FTH1 and FTL may play a role in injury modulation. Our findings highlight macrophage ferritin as a key regulator of macrophage survival and the response of the lung to injury, presenting a potentially targetable pathway for ARDS treatment.

## INTRODUCTION

Acute respiratory distress syndrome (ARDS) is a heterogeneous syndrome that is characterized clinically by rapid onset of respiratory failure and histologically by acute lung injury and diffuse alveolar damage^1, 2^. While both infectious and noninfectious etiologies can precipitate ARDS development, ARDS, at its core, is characterized by abnormal inflammation and alveolar barrier disruption that precipitates an influx of protein- and immune cell-rich edema fluid^1^, impeding gas exchange. Among the immune cells that play a central role in ARDS are lung macrophages^3^, a mixed population of resident alveolar and interstitial macrophages, as well as recruited monocytes. Macrophages are also accompanied by the arrival of neutrophils which migrate to the lung in response to chemotactic signaling and increased epithelial permeability specific to ARDS^4, 5^. Macrophages and monocytes shape the trajectory of ARDS both early in the disease course^5, 6, 7^ and later in lung injury resolution^8, 9^, and have both protective and pathogenic roles in experimental ARDS models^10, 11, 12^.

In addition to orchestrating the immune response, macrophages are master regulators of tissue iron homeostasis^13^. While iron levels vary across different organs, both tissue-resident macrophages and circulating monocytes possess complex iron-handling mechanisms that carefully regulate iron in the extracellular milieu^14^. This regulation is crucial because insufficient intracellular iron disrupts normal cellular function, while excess free intracellular iron can catalyze the formation of oxygen and nitrogen radicals, leading to the production of phospholipid peroxides^15^. These peroxides in turn propagate along biological membranes and trigger a regulated cell death pathway termed ferroptosis^16^. Although the precise roles of these iron-catalyzed processes in ARDS pathogenesis remain unclear^17, 18, 19, 20^, there are observational and experimental data to support a connection between macrophage iron handling and ARDS. Specifically, iron and iron-related proteins are increased in the lower respiratory tract of individuals with ARDS^21^, and macrophage or neutrophil iron loading increases injury in experimental ALI models^22, 23, 24^. One possible way iron may contribute to ARDS pathology involves the iron storage protein ferritin. Intracellular ferritin is a heteropolymer composed of ferritin heavy chain (FTH1) and ferritin light chain (FTL) subunits and is induced by cellular iron loading as a way to sequester intracellular iron in a non-toxic form^25^. FTH1 in particular is important and essential for survival, as demonstrated by the lethal effects of ferritin gene deletion in murine embryos^26, 27^. Systemic loss of FTH1 in adulthood also leads to severe multi-organ damage which is fatal in mice^28^, and mutations in critical FTH1 functional regions are likely to be fatal in humans^29^. Ferritin is also secreted into the extracellular space^30^ where it may function as a short-range cell-to-cell carrier of ferric iron^31, 32, 33, 34^. Extracellular ferritin (ex-ferritin) is likely similarly regulated by iron, is also composed of FTH1 and FTL, and is known to have immunomodulatory and myelopoiesis altering properties^35, 36, 37^. In the setting of ARDS, elevated serum ex-ferritin levels precede clinical deterioration and are linked to adverse outcomes in individuals with ARDS in multiple studies^38, 39, 40, 41, 42, 43^. Nevertheless, the biological role of ex-ferritin in ARDS is unknown, and whether increased ex-ferritin is merely a non-specific feature of inflammation and critical illness or whether there is a mechanistic reason for its production remain undetermined.

In this study, we hypothesized that the main source for ex-ferritin in critical illness is the macrophage and that macrophage ferritin regulation plays a mechanistic role in the dysregulated systemic inflammatory response commonly observed in ARDS. Using three COVID-19 associated ARDS cohorts combined with mechanistic studies in a murine model of acute lung injury, we show that ferritin is central to the response of macrophages to acute lung injury via the regulation of ferroptosis, independent of intracellular iron status. Our results implicate macrophage ferritin as a crucial regulator of immune activation and macrophage survival in ARDS, and suggest that macrophage ferritin metabolism may be an important and potentially targetable mechanistic pathway in ARDS pathogenesis.

## RESULTS

### Serum ferritin trajectory associates with increased mortality in COVID-19 ARDS.

**Figure 1.**
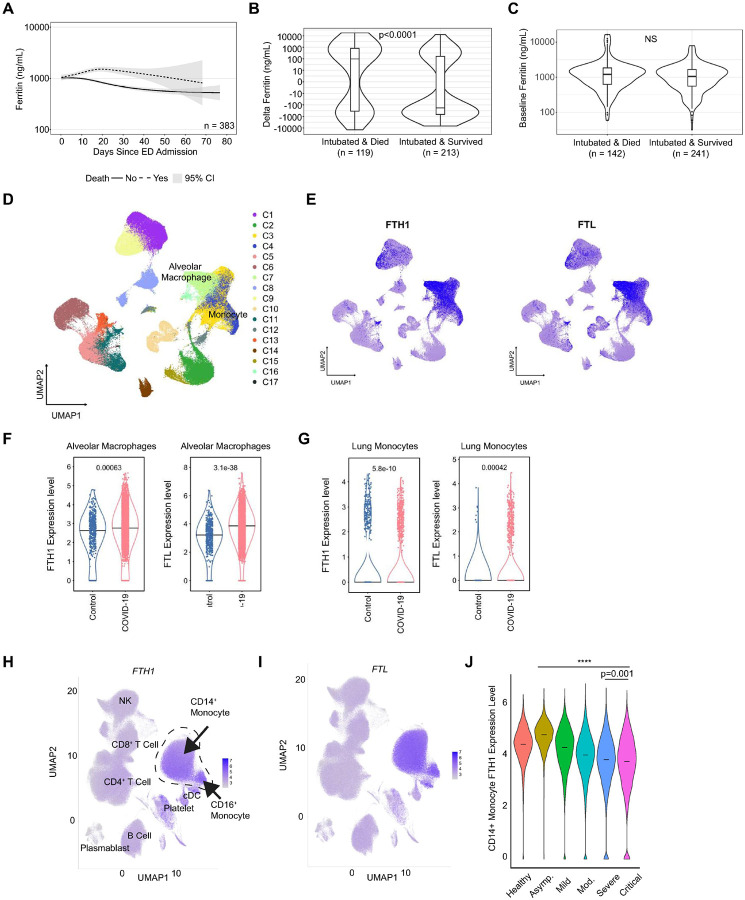
Serum ferritin associates with mortality in patients with COVID-19 ARDS. **(A)** Serum ferritin trajectory in patients who were intubated due to respiratory failure from severe COVID-19 in those who survived (n=241, solid line) and those who died (n=142, dashed line). Delta ferritin, **(B)** defined highest serum ferritin level within 21 days of hospitalization subtracting baseline ferritin, and baseline ferritin **(C),** dichotomized by mortality in the NYP COVID-19 Cohort. Data presented using violin plots with median and box indicating upper and lower quartiles, p-values calculated by Kruskal-Wallis test. **(D)** UMAP representation of *FTH1* and *FTL* **(E)** in main lung cell populations, with alveolar macrophage **(F)** and monocyte **(G)** expression presented as violin plots with medians. UMAP representation feature plot showing main peripheral blood cell types across all donors and expression of *FTH1* **(H)** and *FTL* **(I). (J)** Violin plots of CD14+ Monocytes of *FTH1* expression by donor cells across increasing covid severity. P-values calculated by Kruskal-Wallis test. ****p<0.0001.

We first assessed if increased extracellular ferritin (ex-ferritin) levels correlated with ARDS in a large clinical cohort (n=1408) of individuals hospitalized with COVID-19 at New York Presbyterian (NYP) Weill Cornell Medicine Center and New York Presbyterian Lower Manhattan Hospital. 1102 of these hospitalized individuals had one or more serum ex-ferritin measurements, with 383 requiring mechanical ventilation for respiratory failure due to COVID-19 (**Supplementary Figure 1A, Supplementary Table 1**). On average, patients who were intubated and died from COVID-19 had a rising serum ex-ferritin trajectory during their hospital stay compared to patients who were intubated but survived (Figure 1A). This difference in trajectory can be quantified by comparing delta ex-ferritin, the difference between a patient’s initial ex-ferritin and their last ex-ferritin value before day 21 of hospitalization (as illustrated in **Supplementary Figure 1B**). The median delta ex-ferritin among patients who were intubated and died (n=119) was positive (+96.6 ng/mL), whereas the median delta ex-ferritin who were intubated and survived was significantly negative (−181.1 ng/mL, n=213, *p*<0.0001, Figure 1B), despite the subgroups having similar baseline ex-ferritin upon admission (1209.4 ng/mL died vs. 1055.5 survived, *p*=0.17) (Figure 1C). Individuals who had delta ex-ferritin above the median also had significantly higher 28-day mortality (37%) compared to patients who had delta ex-ferritin below the median (18%, *p*<0.001), as well as fewer ventilator-free days (*p*=0.019), despite having similar ventilator parameters at ICU admission and similar rates of COVID-19 related complications (Table 1). These data show that ex-ferritin levels rise with increasing ARDS disease severity in COVID-ARDS and associate with worse clinical outcomes.

### FTH1 and FTL expression are enriched in monocytes and lung macrophages in individuals with ARDS

The source of serum ex-ferritin in ARDS is unknown, but one of the leading candidates is the macrophage^44, 45^. We thus next examined the association between ferritin and macrophages in the setting of COVID-19^46^ by querying two available single-cell RNASeq (scRNA-Seq) datasets. Using a single-cell lung atlas of severe COVID-19 (containing 7 healthy non-COVID-19 controls who underwent lung resection or biopsy and 19 individuals with lethal COVID-19)^47^, we found that lung monocytes and macrophages had the highest expression of *FTH1* and *FTL* compared to other immune and structural cells in the lung (Figure 1D–E). Lung resident macrophage *FTH1* and *FTL* expression were significantly higher in individuals who died from COVID-19 when compared to healthy controls^48^ (Figure 1F). Conversely, lung monocyte *FTH1* and *FTL* expression were significantly lower in individuals with lethal COVID-19 when compared to healthy controls^48^ (Figure 1G). To validate these contrasting findings, we analyzed an available peripheral blood scRNA-Seq dataset (containing 41 non-COVID-19 controls and 102 COVID-19 patients of varying severity)^46^. Compared to other innate and adaptive immune cells such as NK cells, B cells, and T cells, monocytes (both classical CD14^+^ and nonclassical CD16^+^) had the highest expression of *FTH1* and *FTL* (Figure 1H–I). In CD14^+^ classical monocytes, the expression of *FTH1* increased in asymptomatic COVID-19 patients compared to healthy controls, subsequently declining with increasing disease severity (Bonferroni-adjusted *p*< 0.0001 for all comparisons except for between severe and critical subgroups for FTH1, for which *p*=0.0011, Figure 1J). This decrease was also observed for *FTL* in CD14^+^ monocytes (**Supplementary Figure 2A**), but similar relationships between *FTH1* or *FTL* with disease severity were not seen in the CD16+ monocyte population or other myeloid populations (*data not shown*). A decrease in monocyte *FTH1* expression is also seen in the subset of subjects in the NYP COVID-19 cohort who had blood drawn for bulk RNA Sequencing (RNA-Seq) of peripheral blood mononuclear cells (PBMCs), where we found a trend for lower FTH1 transcripts per million (TPM) in subjects with COVID-19 ARDS (n=48) compared to those with COVID-19 pneumonia (n=6) (**Supplementary Figure 2B**). The above findings reveal that ferritin transcripts are highly enriched in myeloid cells within the lungs and peripheral blood of critically ill COVID-19 patients but show a distinct divergence in expression levels whereby alveolar macrophages exhibit higher ferritin expression, while monocytes display significantly lower levels in individuals with ARDS when compared to healthy controls.

### Hyperoxia-induced ARDS modulates macrophage ferritin expression and extracellular ferritin.

**Figure 2.**
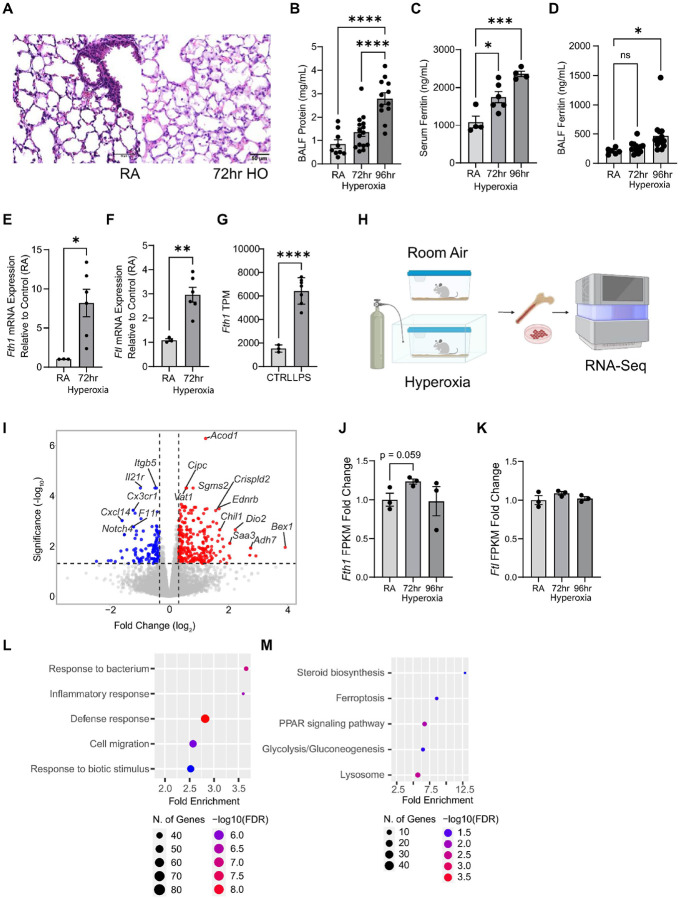
Hyperoxia-induced lung injury (HALI) as a murine ARDS model. **(A)** Representative histology of an affected lung in control mice *(Fth1*^*fl/fl*^*)* following 72 hours of hyperoxia exposure, showing alveolar injury characterized by alveolar epithelial cell degeneration and necrosis, neutrophilic infiltrates in alveolar septum, and fibrillar proteinaceous material in alveolar spaces. Scale bars 50μm. Bronchoalveolar lavage fluid (BALF) protein **(B)**, serum ferritin **(C)**, and BALF ferritin **(D)** with progressive hyperoxia exposure. Bronchoalveolar lavage (BAL) cell mRNA expression of *Fth1* **(E)** and *Ftl* **(F)** from *Fth1*^*fl/fl*^ control mice, relative to RA expression. **(G)** Fth1 Transcripts per Million (TPM) from bulk RNA-Seq of BAL cells following intratracheal LPS instillation. **(H)** Schematic of BMDM RNA-Seq experiment, created using biorender (https://app.biorender.com/). **(I)** Volcano plot highlighting gene expression (n=3 for both groups) changes comparing BMDMs from *Fth1*^*fl/fl*^ mice exposed to 72 hours of hyperoxia compared to those from room air (RA) *Fth1*^*fl/fl*^ mice, using a cut-off of 1.25 fold-change and false discovery rate (FDR) less than 0.05. Figure illustrated using VolcaNoseR (https://goedhart.shinyapps.io/VoicaNoseR/). BMDM expression of *Fth1* **(J)** and *Ftl* **(K)** in *Fth1*^*fl/fl*^ mice from the RA and 72- and 96-hour hyperoxia conditions in Fragments Per Kilobase of transcript per Million mapped reads (FPKM), normalized to RA expression. **(L)** Gene ontology (biological process) and **(M)** Kyoto Encyclopedia of Genes and Genomes (KEGG) analysis of upregulated genes in BMDMs from mice exposed to hyperoxia relative to those from RA mice, illustrated using ShinyGo (http://bioinformatics.sdstate.edu/go/). Data (B-G, J-K) presented with mean ± SEM with p-value by analysis of variance (ANOVA) with Sidak’s correction for multiple comparisons, or by unpaired Student’s t-test, as appropriate. *p<0.05, **P<0.01, ***p<0.001, ****p<0.0001

Given the importance of lung macrophages including monocyte-derived lung macrophages to the pathophysiology of ARDS, we next utilized a murine hyperoxia-induced acute lung injury (HALI) model^49, 50, 51, 52^ to examine ferritin expression in alveolar macrophages (AMs) and monocyte-derived macrophages during lung injury. HALI is a commonly used experimental model for ARDS^53^ whereby prolonged exposure to high oxygen (>95%) results in robust alveolar and bronchiolar epithelial cell injury (Figure 2A). The injury is characterized by alveolar and bronchiolar epithelial cell necrosis and degeneration along with neutrophils and macrophages infiltrating alveolar and peribronchiolar spaces, resulting in increased alveolar capillary permeability characterized by increased bronchoalveolar lavage fluid (BALF) protein (Figure 2B), eventually resulting in mortality starting approximately 72 hours after hyperoxia onset. Notably, in this model, exposure to hyperoxia increased serum ex-ferritin in a time dependent manner (Figure 2C), mirroring the increase in serum ex-ferritin with human ARDS. Similarly, bronchoalveolar lavage fluid (BALF) ferritin also increased upon hyperoxia exposure, suggesting that a similar response is occurring in the lung microenvironment (Figure 2D). To further examine ferritin expression, we isolated RNA from BALF cells (which consist mainly of macrophages) from mice exposed to 72 hours hyperoxia. *Fth1* and *Ftl* mRNA expression were significantly higher in the BALF-derived cells of mice exposed to hyperoxia, compared to those from room air (RA) mice (Figure 2E–F). *Fth1* mRNA expression was also significantly higher but *Ftl* transcripts significantly lower (**Supplementary Figure 2C**) in AMs isolated from mice exposed to intratracheal lipopolysaccharide (LPS, 6 hours), another model of acute lung injury, by bulk RNA-Seq (Figure 2G).

To assess ferritin changes in monocyte-derived macrophages in the HALI model, we isolated bone marrow from mice exposed to 72 or 96 hours of hyperoxia (n=3 in each group and each timepoint) along with RA control mice (Figure 2H). We differentiated bone marrow precursor cells to bone-marrow derived macrophages (BMDMs) *in vitro* using M-CSF, then collected these cells and performed bulk RNA-Seq and analysis. Using cut-offs of false discovery rate (FDR) of 0.05 and 1.25-fold change (log_2_FC=|0.3219|), we found that hyperoxia induced significant gene expression changes in BMDMs, with 132 genes downregulated and 269 upregulated (Figure 2I) in BMDMs from mice exposed to 72 hours of hyperoxia compared to those from RA mice. BMDM *Fth1* mRNA expression was increased following 72 hours of hyperoxia and trended towards a decline with further exposure, while *Ftl* mRNA expression was unchanged (Figure 2J–K), mirroring PBMC *FTH1* expression trajectory seen in human COVID-19 ARDS (Figure 1J). GO (gene ontology) Biological Process analysis indicated that BMDMs from mice exposed to 72 hours of hyperoxia had increased expression of genes associated with response to bacteria, including iron scavenging proteins such lactoferrin (*Ltf*) and lipocalin-2 (*Lcn2*) as well as increased expression of genes related to an activated macrophage responses (inflammatory response, defense response, cell migration) (Figure 2L). Kyoto Encyclopedia of Genes and Genomes (KEGG) analysis revealed upregulated pathways such as ferroptosis, with genes including *Slc7a11*, a critical cysteine importer which defends against ferroptosis, and PPAR signaling, including multiple fatty acid binding proteins *Fabp3*, *Fabp4*, and *Fabp5*. (Figure 2M). Similar gene expression differences were found comparing BMDMs from control mice and mice exposed to 96 hours of hyperoxia, with 192 genes downregulated and 191 upregulated and comparable pathways were enriched (**Supplementary Figure 3A-B**). These findings indicate that hyperoxia significantly elevates FTH1 expression, and to a lesser extent FTL, in AMs and in monocyte-derived macrophages. This increase is associated with an activated macrophage signature and a rise in serum and BALF ex-ferritin levels in a murine HALI model, mirroring patterns observed in human ARDS.

### Macrophage FTH1 deficiency protects against hyperoxia-induced lung injury.

**Figure 3.**
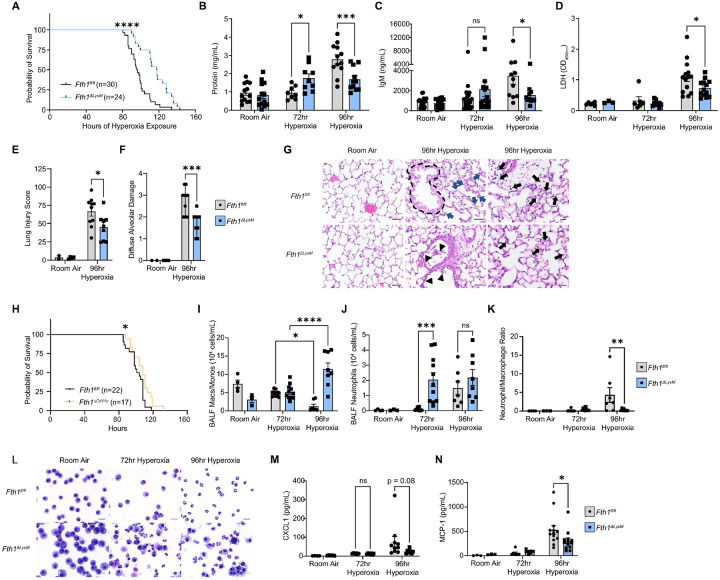
Myeloid FTH1-deficient mice are protected against hyperoxia-induced acute lung injury. **(A)** Probability of survival of *Fth1*^*fl/fl*^ and *Fth1*^*ΔLysM*^ mice in hyperoxia with survival analysis p-value by Gehan-Breslow-Wilcoxon test. BALF protein **(B**, n=13/8/12 for *Fth1*^*fl/fl*^ mice and n=13/9/11 for *Fth1*^*ΔLysM*^ mice at the room air/72hr/96hr time points), IgM **(C**, n=21/23/11 for *Fth1*^*fl/fl*^ mice and n=17/18/11 for *Fth1*^*ΔLysM*^ mice at the room air/72hr/96hr time points), and LDH **(D**, n=6/6/14 for *Fth1*^*fl/fl*^ mice and n=3/9/13 for *Fth1*^*ΔLysM*^ mice at the room air/72hr/96hr time points). **(E-F)** Histological lung injury at 96-hours measured by the Lung Injury Score presented as median with 95% confidence interval, p-values by unpaired Student’s t-test. *p<0.05, ***p<0.001. **(G)** Representative hematoxylin and eosin (H&E) staining of lung sections from mice exposed to hyperoxia or room air for 96 hours (n=2 *Fth1*^*fl/fl*^ mice and n=4 *Fth1*^*ΔLysM*^ mice in room air, n=10 *Fth1*^*fl/fl*^ mice and n=9 *Fth1*^*ΔLysM*^ mice in hyperoxia). Dotted line highlights marked diffuse epithelial necrosis in a bronchiole with adjacent areas of alveolar epithelial cell necrosis and septal wall hyalinization (blue arrows). Arrowheads draw attention to multifocal foci of bronchiolar epithelial attenuation and necrotic debris in the lumen of a bronchiole. Black arrows indicate neutrophils in the alveolar septum and perivascular space. Scale bars indicate 40 μm in left and middle panels and 60 μm in right panels. **(H)** Probability of survival of *Fth1*^*fl/fl*^ and *Fth1*^*ΔCd11c*^ mice in hyperoxia. with p-value calculated by Gehan-Breslow-Wilcoxon test. *p<0.05. BALF macrophages and monocytes **(I**, n=4/12/6 for *Fth1*^*fl/fl*^ mice and n=4/11/6 for *Fth1*^*ΔLysM*^ mice at the room air/72hr/96hr time points) and neutrophils **(J**, n=4/12/6 for *Fth1*^*fl/fl*^ mice and n=4/11/6 for *Fth1*^*ΔLysM*^ mice at the room air/72hr/96hr time points) cell numbers, and neutrophil/macrophage ratio **(K**, n=4/12/8 for *Fth1*^*fl/fl*^ mice and n=4/11/8 for *Fth1*^*ΔLysM*^ mice at the room air/72hr/96hr time points) by manual counting of cytospin slides made from lavage cells from *Fth1*^*fl/fl*^ and *Fth1*^*ΔLysM*^ mice in room and 72- and 96-hour hyperoxia conditions, with representative 40x magnification images in **(L)**, scale bars indicate 20 μm. BALF CXCL1 **(M**, n=4/8/10 for *Fth1*^*fl/fl*^ mice and n=5/6/7 for *Fth1*^*ΔLysM*^ mice at the room air/72hr/96hr time points) and MCP-1 **(N**, n=3/7/13 for both *Fth1*^*fl/fl*^ and *Fth1*^*ΔLysM*^ mice at the room air/72hr/96hr time points). Data (B-D, l-K, M-N presented as mean ± SEM, p-values by 2-way analysis of variance (ANOVA) with Šídák’s correction for multiple comparisons. *p<0.05, **p<0.01, ***p<0.001, ****p<0.0001.

The observation that AM and BMDM *Fth1* expression was robustly upregulated in our hyperoxia model mirrored our findings of *FTH1* upregulation in lung macrophages in human COVID-19 ARDS. To examine the mechanistic role of FTH1 in ARDS, we generated a murine model of FTH1 depletion in myeloid cells using Cre-Lox recombination, placing *Fth1* under the control of the *Lyz2* promoter (*Fth1*^*ΔLysM*^, **Supplementary Figure 4A**). Targeted *Fth1* depletion was confirmed using RT-qPCR and immunoblotting in both BMDMs as well as AMs (**Supplementary Figure 4B-D**). We first examined the response of *Fth1*^*fl/fl*^ and *Fth1*^*ΔLysM*^ mice to hyperoxia. *Fth1*^*ΔLysM*^ mice consistently survived longer in hyperoxia than littermate *Fth1*^*fl/fl*^ control mice, with a median survival of 118 hours vs 96 hours (*p*<0.0001, Figure 3A). Pathological assessment of *Fth1*^*ΔLysM*^ mice at 96 hours of hyperoxia exposure revealed that this difference in survival was associated with decreased lung injury, with no evidence of differences in tissue injury in other organs such as the heart or the brain upon necropsy (*not shown*). Consistently, *Fth1*^*ΔLysM*^ micealso had lower BALF protein and IgM compared to *Fth1*^*fl/fl*^ mice exposed to 96 hours of hyperoxia, reflecting protection from alveolar capillary permeability Figure 3B–C). Additionally, *Fth1*^*ΔLysM*^ micehad lower BALF lactate dehydrogenase (LDH) relative to *Fth1*^*fl/fl*^ mice exposed to 96 hours of hyperoxia, suggestive of reduced cell death in the lung (Figure 3D). These surrogate lung injury markers were consistent with histopathological changes in the lung, which showed decreased bronchiolar and alveolar epithelial cell necrosis and neutrophilic infiltrates in the alveoli and interstitial spaces as well as decreased histological diffuse alveolar damage in hyperoxia-exposed *Fth1*^*ΔLysM*^ mice when compared to *Fth1*^*fl/fl*^ mice, as quantified by a standard acute lung injury scoring systems^53, 54^ (Figure 3E–F, representative images in Figure 3G). These findings were recapitulated and validated in mice with a targeted deletion of FTH1 in the resident alveolar macrophage population using a Cd11c-Cre driver^55^ whereby compared to littermate control *Fth1*^*fl/fl*^ mice, *Fth1*^*ΔCd11c*^ mice had improved survival (Figure 3H). These results demonstrate that macrophage FTH1 modulates lung injury and mortality arising from hyperoxia-induced lung injury.

### Macrophage FTH1 depletion alters the cellular inflammatory response to hyperoxia.

To examine how macrophage FTH1 drives lung injury upon hyperoxia exposure, we next examined the effect of macrophage FTH1 depletion on the immune response to hyperoxia and on hyperoxia-induced cell injury. In addition to macrophages, neutrophils play a significant role in ARDS development^56^. In human ARDS, the number of neutrophils found in BAL fluid is associated with the severity of gas exchange and lung protein permeability^57^ as well as BAL inflammatory cytokine levels and degree of lung injury^58^. Thus, we first quantified the amount of BAL macrophages and BAL neutrophils that infiltrated the airways upon hyperoxia using manual differential counting on BALF cell cytospin preparations. *Fth1*^*ΔLysM*^ mice, which had increased survival under hyperoxia displayed an early onset of macrophage and neutrophil infiltration in the airspaces at 72 hours when compared to *Fth1*^*fl/fl*^ controls (Figure 3I–J). This early onset of neutrophil infiltration in the *Fth1*^*ΔLysM*^ mice at 72 hours mirrored higher BALF protein (*p*=0.04) and a trend for higher BALF IgM at 72 hours compared to *Fth1*^*fl/fl*^ mice (Figure 3D–E). However, at 96 hours, *Fth1*^*ΔLysM*^ mice had significantly higher numbers of macrophages compared to *Fth1*^*fl/fl*^ controls, whereas in contrast neutrophils were the dominant airway immune cell found in the *Fth1*^*fl/fl*^ control mice (Figure 3I–J). This neutrophil-macrophage imbalance is represented by a lower neutrophil to macrophage ratio in *Fth1*^*ΔLysM*^ mice when compared to the *Fth1*^*fl/fl*^ mice at 96 hours (Figure 3K, representative images Figure 3L) and is associated with lower lung injury markers such as BALF protein and IgM (Figure 3D–E). Similar trends in BAL differential cell counts were also observed in *Fth1*^*ΔCd11c*^ mice exposed to hyperoxia (**Supplementary Figure 5A-B**). At the 96-hour timepoint, BALF from *Fth1*^*ΔLysM*^ mice had lower levels of CXCL1, the principal neutrophil chemoattractant, as well as lower levels of macrophage/monocyte chemoattracts MCP-1 (CCL2), MCP-3 (CCL7), and MCP-5 (CCL12), compared to *Fth1*^*fl/fl*^ mice (Figure 3M–N, **Supplementary Figure 6A-B**). These results suggest that the lower airway macrophage and monocyte numbers in control *Fth1*^*fl/fl*^ relative to that of *Fth1*^*ΔLysM*^ mice may not be due to the absence of chemotactic signals.

### FTH1 depletion protects macrophages from ferroptosis.

**Figure 4.**
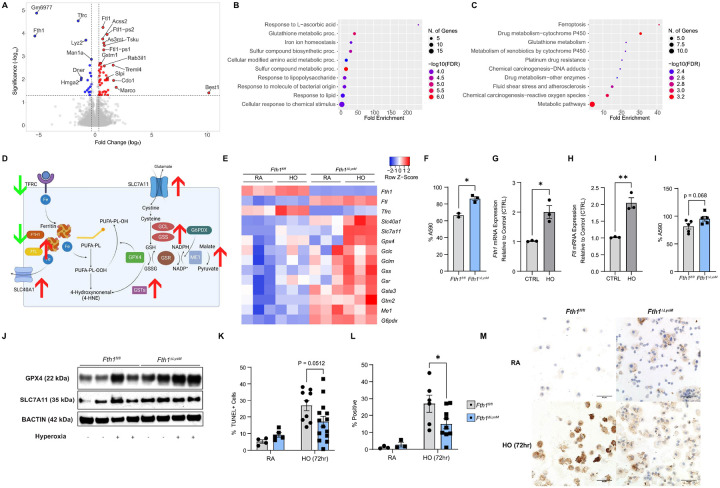
Myeloid FTH1-defiency protects against ferroptosis. **(A-F)** Volcano plot highlighting gene expression (n=3 for all groups) changes comparing BMDMs from *Fth1*^*fl/fl*^ and *Fth1*^*ΔLysM*^ *mice* exposed to 72 hours of hyperoxia **(A)** using a cut-off of 1.25 fold-change and FDR) < 0.05; illustrated using VolcaNoseR. Upregulated genes analyzed using Gene Ontology **(B)** Biological Process and **(C)** KEGG are illustrated using ShinyGo. **(D-E)** Schematic of critical proteins involved in iron-handling and ferroptosis, with their expression in BMDMs from *Fthf*^*fl/fl*^ and *Fth1*^*ΔLysU*^*mice* from room air and hyperoxia, illustrated using Heatmapper (http://www.heatmapper.ca/expression/). **(F)** Viability as measured by the Presto Blue Assay in BMDMs from *Fth1*^*fl/fl*^ and *Fth1*^*ΔLysM*^ mice 4 hours after treatment with 1 μM RSL3, relative to cells treated with DMSO. mRNA expression of *Fth1* **(G)** and *Ftl* **(H)** in *Fth1*^*fl/fl*^ and *Fth1*^*ΔLysM*^ BMDMs exposed to in vitro hyperoxia. **(I)** Viability as measured by Presto Blue of *Fth1*^*fl/fl*^ *and Fth1*^*ΔLysM*^ *BMDMs* exposed to 24 hours of in vitro hyperoxia as percentage of absorbance values of matched control cells. **(J)** Immunoblot of GPX4 and SLC7A11 protein expression in bronchoalveolar lavage (BAL) cells from *Fth1*^*fl/fl*^ and *Fth1*^*ΔLysM*^ mice from RAand hyperoxia conditions (n=2 in each group). **(K)** Total TUNEL+ immune cells on cytospin slides made from lavage cells from *Fth1*^*fl/fl*^ and *Fth1*^*ΔLysM*^ mice in room and 72-hour hyperoxia condition (n=4/9 for *Fth1*^*fl/fl*^ mice and n=5/14 for*Fth1*^*ΔLysM*^ mice at the room air and hyperoxia time points). **(L)** 4-hydroxynonenal staining by immunocytochemistry on BAL cell cytospins from *Fth1*^*fl/fl*^ and *Fth1*^*ΔLysM*^ mice under room air and following hyperoxia exposure with representative images **(M)**. Scale bars indicate 50 μm. Data (F-l, K-L) presented as mean ± SEM, p-values by unpaired Student’s t-test (F-l) and 2-way analysis of variance (ANOVA) with Šídák’s correction for multiple comparisons (K-L). *p<0.05, **p<0.01.

Given these findings, we next explored whether the increased abundance of airspace macrophages and the lower neutrophil:macrophage ratios in the lungs of *Fth1*^*ΔLysM*^ mice under hyperoxia is due to increased macrophage survival. Alveolar macrophage cell death plays an important role in response of the lung to injury with macrophage inflammation and cell death mutually influencing each other to create a self-amplifying loop that exacerbates inflammation^59, 60, 61^. To assess if protection from macrophage cell death is playing a role in the lower neutrophil: macrophage ratios observed in the *Fth1*^*ΔLysM*^ mice under hyperoxia, we isolated bone marrow from *Fth1*^*fl/fl*^ and *Fth1*^*ΔLysM*^ mice exposed to room air or hyperoxia (n=3 in each group and each timepoint), and generated BMDMs *in vitro*. Bulk RNA-Seq analysis using cut-offs of false discovery rate (FDR) of 0.05 and 1.25-fold change (log_2_FC 0.3219) revealed 63 differentially expressed genes in macrophages harvested from *Fth1*^*fl/fl*^ mice exposed to 72 hours of hyperoxia compared to those from *Fth1*^*ΔLysM*^mice exposed to 72 hours of hyperoxia (19 down and 44 up, Figure 4A). Aside from a decrease in *Fth1* expression, at baseline BMDMs from *Fth1*^*ΔLysM*^mice had increased expression of genes involved in glutathione metabolism and defense against ferroptosis, an iron-catalyzed cell death pathway^16^, both via GO analysis (Biological Processes, Figure 4B) and in pathway analysis via KEGG (Figure 4C). Similar differentially expressed genes and pathways were found compared macrophages from *Fth1*^*fl/fl*^ and *Fth1*^*ΔLysM*^mice at room air (**Supplementary Figure 7A-C**) and following 96 hours of hyperoxia exposure (**Supplementary Figure 7D-E**), suggesting that these differences were largely drive by FTH1 depletion.

Ferroptosis is a form of regulated cell death that involves iron-catalyzed generation of membrane lipid peroxides. In our data, comparing FPKM (fragments per kilobase of exon per million mapped fragments) at the individual gene level (Figure 4D–E), FTH1-deficient BMDMs showed increased expression of *Ftl* and the iron exporter ferroportin (*Slc40a1*) but decreased expression of transferrin receptor (*Tfrc*), indicating that these macrophages may be releasing excess iron and limiting the uptake of iron, both of which are protective against ferroptosis. Furthermore, iron catalyzes the formation of lipid peroxides which are the ultimate executors of ferroptosis. The expression of two key proteins that safeguard against these peroxides, glutathione peroxidase 4 (*Gpx4*), which reduces lipid peroxides to harmless alcohols using glutathione, and *Slc7a11* (also known as system Xc^−^ or xCT), a glutamate/cystine antiporter which is essential for glutathione synthesis, are both significantly increased in hyperoxia and in *Fth1*^*ΔLysM*^BMDMs (Figure 4D–E). We also examined the glutathione synthesis pathway downstream of SLC7A11 and found increased transcriptomic expression of glutamate–cysteine ligase (*Gclc*), glutathione synthetase (*Gss*), glutathione reductase (*Gsr*), as well as several glutathione S-transferases (GSTs) which act on peroxidation products, in *Fth1*^*ΔLysM*^ BMDMs. Glucose-6-phosphate dehydrogenase (*G6pdx*) and malic enzyme (*Me1*), which replenishes the NAPDH used to generate glutathione, were also upregulated in the *Fth1*^*ΔLysM*^ BMDMs when compared to control *Fth1*^*fl/fl*^ BMDMs. These findings show that FTH-1 deficient macrophages have altered iron homeostasis and upregulate iron- and glutathione-independent mechanisms that may directly counteract ferroptosis.

We validated these expression differences in iron and anti-ferroptosis genes *in vitro* and *in vivo*. *Fth1*^*ΔLysM*^ BMDMs have improved survival compared to *Fth1*^*flfl*^ BMDMs following treatment with rasselective lethal small molecule 3 (RSL3), a chemical ferroptosis inducer, as quantified by the Presto Blue Assay (Figure 4F). *In vitro,* hyperoxia induces BMDM *Ftl* and *Fth1* mRNA expression (Figure 4G–H), similar to what is observed *in vivo* model, with *Fth1*^*ΔLysM*^ BMDMs being more resistant to *in vitro* hyperoxia-induced cell death compared to *Fth1*^*flfl*^ cells (Figure 4I). We assessed for similar changes in BAL cells in *Fth1*^*fl/fl*^ and *Fth1*^*ΔLysM*^ from mice exposed to room air or hyperoxia conditions and found that GPX4 and SLC7A11 protein levels were higher in BAL cells collected from *Fth1*^*ΔLysM*^ mice in room air and increase further following 72 hours of hyperoxia exposure when compared to BAL cells from *Fth1*^*fl/fl*^ mice (Figure 4J). At the 72-hour timepoint, *Fth1*^*fl/fl*^ mice also had increased numbers of TUNEL+ cells compared to *Fth1*^*ΔLysM*^ mice indicating more immune cell death in the *Fth1*^*fl/fl*^ mice (Figure 4K). BAL immune cells from *Fth1*^*ΔLysM*^ mice following hyperoxia exposure also had decreased immunohistochemical staining of 4-hydroxynoneal (4-HNE), a lipid peroxidation byproduct from ferroptosis, when compared to those from *Fth1*^*fl/fl*^ mice (Figure 4L, representative images in Figure 4M). Taken together the above data suggests that macrophage FTH1 may regulate macrophage ferroptosis in response to hyperoxia and may be one mechanism by which FTH1 deficient macrophages are protected from cell death in hyperoxia-induced experimental ARDS.

### The intracellular iron chelator deferiprone exacerbates hyperoxia-induced lung injury

**Figure 5.**
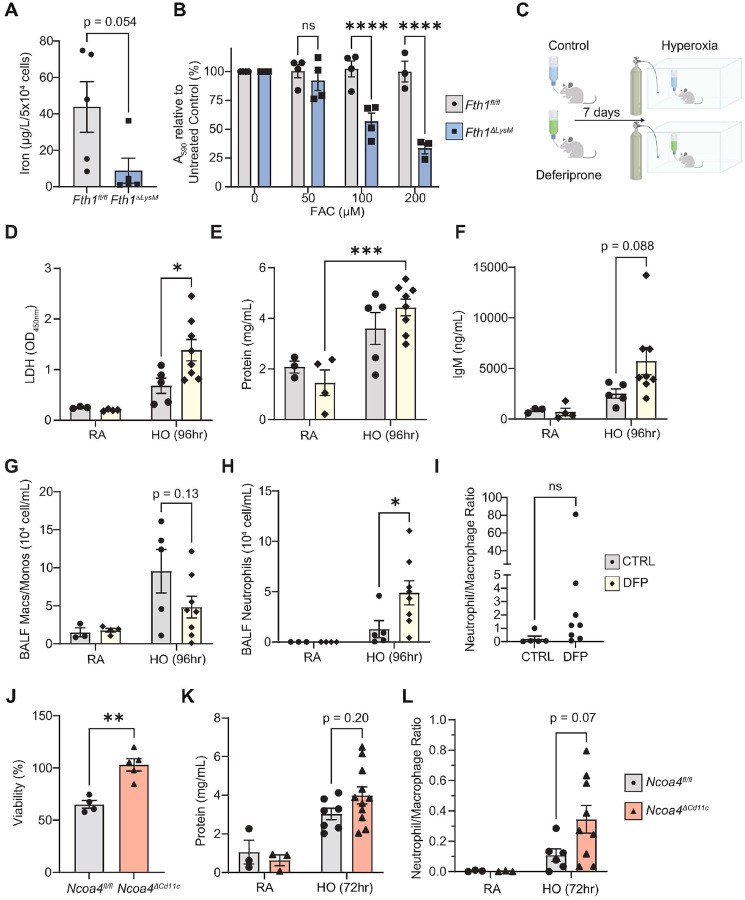
Myeloid FTH1 deficiency alters intracellular and extracellular iron regulation. **(A)** Total cellular iron, normalized to cell count, in *Fth 1*^*fl/fl*^ (n=5) and *Fth1*^*ΔLysM*^ mice (n=5) BMDMs. **(B)** Viability as measured byAlamar Blue and presented byA590 of *Fth1*^*fl/fl*^ *and Fth1*^*ΔLysM*^ BMDMs treated with ferric ammonium citrate (FAC) at 0, 50 μM, 100 μM and 200 μM concentrations for 24 hours compared to untreated cells, n=4 for all groups except for n=3 at the 200 μM FAC concentration. **(C)** Schematic of deferiprone (DFP) hyperoxia experiment, created using biorender. BALF LDH **(D),** protein **(E),** IgM **(F)** in wildtype mice untreated and given deferiprone (DFP) in RA and hyperoxia (HO) conditions, n=3/4/5/8 in untreated RA, DFP RA, untreated HO, DFP HO groups for all experiments. BALF macrophage/monocyte count **(G),** neutrophil count **(H),** and neutrophil/macrophage ratio **(I)** in untreated and DFP-treated mice exposed to 96 hours of hyperoxia. **(J)** Viability as measured byAlamar Blue and presented by A590 of AMs from *Ncoa4*^*fl/fl*^ and ***Ncoa***^***ΔCd11c***^ mice treated with 1 μM RSL4 for 4 hours, relative to untreated cells. BALF protein **(K)** and neutrophil/macrophage ratio **(L)** in *Ncoa4*^*fl/fl*^ and *Ncoa*^*ΔCd11c*^ mice in RAand hyperoxia conditions. Data (A-B, D-L) presented as mean ± SEM with p-values by unpaired Student’s t-test (A-B, J) or 2-way analysis of variance (ANOVA) with Šídák’s correction for multiple comparisons (D-l, K-L), as appropriate. *p<0.05, ***p<0.001, ****p<0.0001.

Ferroptosis is catalyzed by intracellular iron and can be curtailed with the use of ion chelators^16, 62^. To test the hypothesis that intracellular iron availability modulates hyperoxia-induced injury *in vivo*, we first measured total iron levels in BMDMs from *Fth1*^*fl/fl*^ and *Fth1*^*ΔLysM*^ mice. FTH1-deficient BMDMS had significantly lower total iron levels (Figure 5A) measured by graphite furnace atomic absorption spectrometry (GFAAS) when compared to *Fth1*^*fl/fl*^ controls. We postulated that this strict iron regulation is necessary because FTH1-deficient BMDMs cannot store toxic free iron. This is supported by our RNA-Seq data, with *Fth1*^*ΔLysM*^BMDMs having decreased expression of transferrin receptor (*Tfrc*) and increased expression of ferroportin (*Slc40a1*), which limit iron uptake and increase iron release, respectively (Figure 4D–E). This hypothesis is further supported by our observation that *Fth1*^*ΔLysM*^BMDMs are sensitive to exogenous iron as iron added in the form of ferric ammonium citrate (FAC) rapidly decreases their viability relative to *Fth1*^*fl/fl*^ cells over 24 hours (Figure 5B). We speculated that this difference in handling may be the reason why these FTH1-deficient, iron deficient, macrophages are resistant to iron-catalyzed cell death via ferroptosis. To test this hypothesis, we administered the intracellular iron chelator deferiprone (DFP) systemically (1 mg/mL in drinking water) to wildtype C57/BL6 mice 1 week prior to and for the duration of hyperoxia exposure (Figure 5C). DFP reduces macrophage iron *in vitro* (**Supplementary Figure 8**) and reduces systemic iron in as short as 11–14 days in various animal organ injury models^63, 64, 65^. Here, DFP-treated mice developed more severe lung injury in response to hyperoxia, with a significant increases in BALF LDH and a trend for increase in BALF protein and IgM (Figure 5D–F). DFP-treated mice also had decreased absolute BALF macrophage and monocyte counts and increased neutrophils (Figure 5G–H), resulting in an increased neutrophil:macrophage ratio (Figure 5I).

### Impaired macrophage ferritin recycling exacerbates hyperoxia-induced ALI

We next examined the response of mice that cannot liberate bioavailable iron from ferritin breakdown to hyperoxia. Ferritin degradation occurs via a selective autophagic process termed ferritinophagy, and is mediated by the autophagy adaptor protein nuclear receptor coactivator 4 (NCOA4). NCOA4 is increased by intracellular iron depletion^68^, and in turn binds to FTH1 and chaperones it to the autophagosome^66, 67^. Consistent with our findings that FTH1 deficient macrophages have lower bioavailable iron, NCOA4 levels are markedly elevated in FTH1-deficient BMDMs when compared to *Fth1*^*fl/fl*^ mice (**Supplementary Figure 9A**). NCOA4 is also a major regulator of iron recycling in macrophages. NCOA4-deficient BMDMs display impaired iron recycling capacity *in vivo* andfail to degrade ferritin and export iron under conditions of iron deficiency^69, 70^. *In vitro* loss of NCOA4 is protective against chemically-triggered ferroptosis^67^, and *in vivo* disruption of the NCOA4-FTH1 interaction ameliorates ferroptosis-mediated ischemia-reperfusion injury^71^. We thus next generated mice with a targeted deletion of NCOA4 in lung macrophages and examined their response to hyperoxia-induced lung injury, hypothesizing that AM NCOA4 depletion would protect these macrophages from hyperoxia-induced ferroptosis and ameliorate lung injury. Using Cd11c-Cre, we depleted *Ncoa4* in lung macrophages *in vivo,* confirming loss of *Ncoa4* in AMs via qPCR (**Supplementary Figure 9B**). AMs from *Ncoa4*^*ΔCd11c*^ mice had significantly higher *Tfrc* levels and a trend for higher *Fth1* (p=0.07) expression (**Supplementary Figure 9C-D**). AMs from *Ncoa4*^*ΔCd11c*^ mice had similar total iron levels when compared to those from *Ncoa4*^*fl/fl*^ mice (**Supplementary Figure 9E**). NCOA4-deficient AMs were protected from RSL3-induced ferroptosis *in vitro*, confirming the role of NCOA4-mediated ferritinophagy in contributing the necessary iron for ferroptosis (Figure 5J). Contrary to our hypothesis, *Ncoa4*^*ΔCd11c*^ mice developed more lung injury compared with *Ncoa4*^*fl/fl*^ mice when exposed to 72h of hyperoxia with a trend for higher BALF protein (Figure 5K), increased BALF neutrophils, a higher BALF neutrophil:macrophage ratio (Figure 5L and **Supplementary Figure 9F-G**). These results show that impairing AM ferritinophagy and protecting against AM ferroptosis does not protect mice from hyperoxia induced lung injury, suggesting that there may be alternative mechanisms by which *Fth1*^*ΔLysM*^ mice are protected from HALI. Taken together, these results show while FTH1-deficient macrophages have less bioavailable iron, and that pharmacologically restricting iron *in vivo* or impairing AM ferritinophagy to limit iron availability for ferroptosis does not protect mice from hyperoxia-induced lung injury but exacerbates this injury.

### Loss of macrophage FTH1 results in lower extracellular iron and increased FTL ex-ferritin which is protective against hyperoxia

**Figure 6.**
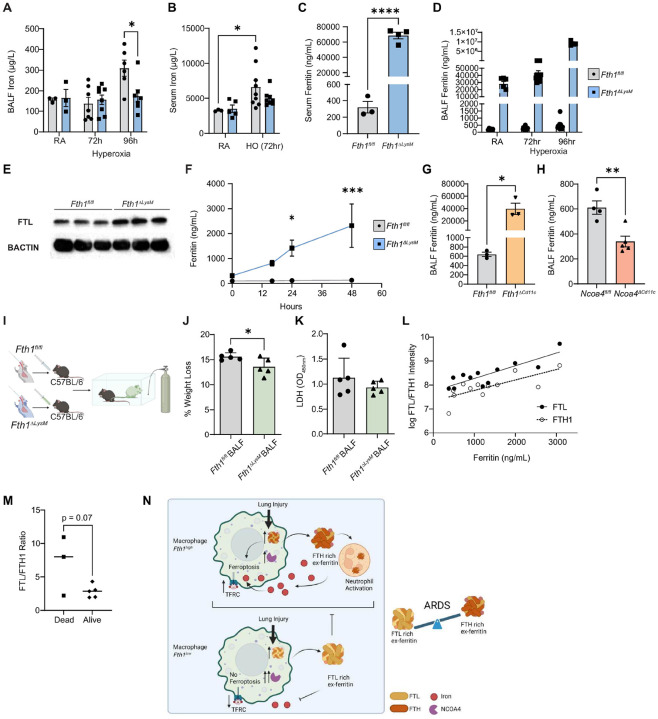
Extracellular ferritin controls extracellular iron levels and is protective against hyperoxia. Total iron in BALF **(A)** and serum **(B)** of *Fth1*^*fl/fl*^ and *Fth1*^*ΔLysM*^ mice at room air (n=3 for *Fth1*^*fl/fl*^ BALF, n=3 for *Fth1*^*fl/fl*^ serum, n=3 for *Fth1*^*ΔLysM*^ BALF, n=5 for *Fth1*^*ΔLysM*^ serum), following 72 hours of hyperoxia (n=7 for *Fth1*^*fl/fl*^ BALF, n=9 for *Fth1*^*fl/fl*^ serum, n=8 for *Fth1*^*ΔLysM*^ BALF, n=8 for *Fth1*^*ΔLysM*^ serum), and following 96 hours of hyperoxia (n=6 for *Fth1*^*fl/fl*^, n=7 for *Fth1*^*ΔLysM*^). Serum ferritin **(C,** n=3 for *Fth1*^*fl/fl*^, n=4 for *Fth1*^*ΔLysM*^*),* and BALF ferritin **(D,** n=6/12/14 for *Fth1*^*fl/fl*^, n=6/14/5 for *Fth1*^*ΔLysM*^ at RA/72hr/96hr hyperoxia conditions) in *Fth1*^*fl/fl*^ and *Fth1*^*ΔLysM*^ in RA and hyperoxia conditions. **(E)** Representative western blot of FTL in untreated *Fth1*^*fl/fl*^ and *Fth1*^*ΔLysM*^ BMDMs. **(F)** Ferritin measured via ELISA in media supernatant in culture of *Fth1*^*fl/fl*^ (n=4) and *Fth1*^*ΔLysM*^ (n=4) BMDMs over time. BALF ferritin in *Fth1*^*fl/fl*^ and *Fth1*^*ΔCd11c*^ mice **(G,** n=3 for both groups) and in *Ncoa4*^*fl/fl*^ and *Ncoa*^*ΔCd11c*^ mice (H, n=4 for *Ncoa4*^*fl/fl*^, n=5 for *Ncoa*^*ΔCd11c*^) at baseline. **(I)** Schematic of BALF transplant experiment, created using biorender. **(J)** Percent weight loss and **(K)** BALF LDH levels in C57BL/6 mice transplanted with BALF from *Fth1*^*fl/fl*^ (n=5) and *Fth1*^*ΔLysM*^ (n=5) mice. **(L)** Ferritin heavy chain (FTH1) and light chain (FTL) intensity relative to serum ferritin from the same subjects, with FTL/FTH1 ratio stratified by patient survival **(M).** Data (A-G, l-K, M-N) presented as mean ± SEM, with p-values calculated by unpaired Student’s t-test (A,, F, l-K, M-N) or 2-way analysis of variance (ANOVA) with Šídák’s correction for multiple comparisons (B-E, G) as appropriate.*p<0.05, **p<−0.01, ***p<0.001, ****p<0.0001. **(N)** Overview schematic of the study.

Ferritin is predominantly known as an intracellular means of storing iron but is also known to be able to transport iron extracellularly^72^. We next assessed if changes in extracellular iron availability are linked to the protection from ferroptosis upon hyperoxia exposure in the *Fth1*^*ΔLysM*^ mice. By GFAAS, extracellular iron levels were significantly lower in the BALF of *Fth1*^*ΔLysM*^ mice when compared to *Fth1*^*fl/fl*^ mice 96 hours after exposure (Figure 6A). Serum iron levels also trended to be lower in the *Fth1*^*ΔLysM*^ mice when compared to *Fth1*^*fl/fl*^ mice 72 hours after hyperoxia exposure (Figure 6B). As mentioned, ferritin is a heteropolymer cage consisting of FTH1 and FTL chain subunits^25^. While the two subunits are structurally similar, they are functionally distinct: whereas FTH1 contains the ferroxidase center which converts Fe^2+^ to Fe^3+^, an essential step for iron incorporation into ferritin, FTL increases the storage capacity or iron binding capacity of ferritin^73^. Ferritin’s function therefore depends on the relative proportion of FTH1 and FTL, which differs in a tissue- and organ-specific manner^74^. Serum ex-ferritin represents a specific form of extracellular ferritin, and is previously thought to originate from damaged cells having lost or liberated most of their iron, with the iron-free protein portion of ferritin assumed to be benign^30^. We therefore next assessed if ex-ferritin levels were different in in the *Fth1*^*ΔLysM*^ mice when compared to controls. Significantly higher serum ex-ferritin levels were detected by enzyme-linked immunosorbent assays (ELISA) in *Fth1*^*ΔLysM*^ mice at baseline, with *Fth1*^*ΔLysM*^ mice having over 1000-fold higher serum ex-ferritin (Figure 6C). We observed the same phenomenon locally in the lung microenvironment with BALF ex-ferritin levels also significantly higher in *Fth1*^*ΔLysM*^ mice when compared to controls and in response to hyperoxia (Figure 6D). We speculated that this increase in ex-ferritin likely originates from macrophages as BMDMs isolated from the *Fth1*^*ΔLysM*^ had higher *Ftl* expression (Figure 6E) and in culture, secreted ferritin, presumably FTL, extracellularly in a time dependent manner (Figure 6F). *Fth1*^*ΔCd11c*^ mice had similarly elevated BALF FTL levels at baseline (Figure 6G), while in direct contrast, *Ncoa4*^*ΔCd11c*^ mice showed decreased BALF ex-ferritin levels (Figure 6H). The above data suggests that upon loss of FTH1 in macrophages, FTL is increased and secreted into the extracellular space and this is associated with lower extracellular iron levels during hyperoxia exposure.

We hypothesized that in the context of elevated serum ex-ferritin in human ARDS, ex-ferritin, specifically FTL, arises from cells that have undergone ferroptosis, and thus not only represents a marker of cellular damage but a way to curb extracellular iron levels to protect from further ferroptosis. Consistent with this hypothesis, extracellular FTL is protective against ferroptosis in models varying from lung adenocarcinoma to pre-eclampsia^75, 76^ and pre-treatment of BMDMs with recombinant FTL attenuates the induction of inflammatory markers in BMDMs^77^. Increased ex-ferritin along with low serum iron is linked to severe COVID-19^78, 79^, although whether this is a maladaptive response that drives disease severity or is an adaptive one that ultimately fails is unclear. To test the hypothesis that a rise in FTL-ex-ferritin confers protection in the setting of hyperoxia-induced lung injury, we isolated BALF from *Fth1*^*fl/fl*^ and *Fth1*^*ΔLysM*^ mice and after centrifugation to remove BAL cells, intratracheally transplanted the supernatant fluid into C57/BL6 mice via intratracheal instillation, 2 hours before exposing to hyperoxia (Figure 6I). After 72 hours of hyperoxia, mice receiving BALF (FTL-ex-ferritin high) from *Fth1*^*ΔLysM*^ mice had decreased weight loss compared to mice receiving BALF from *Fth1*^*fl/fl*^ mice (FTL-ex-ferritin low) (Figure 6J) and a trend for decreased BALF LDH (Figure 6K). The above findings suggest that that in response to a loss of FTH1 macrophages up-regulate and secrete FTL into the extracellular space, with higher FTL levels in the extracellular space associating with lower extracellular iron levels and protection from HALI.

### Serum ex-ferritin consists predominantly of FTL in human ARDS.

Serum ferritin in health is comprised almost exclusively of FTL and is generally thought to be iron poor^35^, but the makeup of serum ferritin in critical illness has yet to be examined. Given our above findings that FTH1 is increased in human and murine macrophages in ARDS, and that mice who have little macrophage FTH1 are protected from ferroptosis under hyperoxia likely due to the increased secretion of FTL which sequesters extracellular iron, we sought to determine the molecular composition of the increased serum ferritin in human COVID-19. We performed mass spectrometry on serum samples from subjects in our COVID-19 cohort (n=12). Taking advantage of ferritin’s stability at high temperatures^80^, we heated serum samples to denature other proteins, thereby enriching serum for ferritin. We then isolated ferritin using native gel electrophoresis, whereupon under the guidance of a native gel ladder and liver ferritin standard, we excised two Coomassie blue-stained bands within the kDa range of native polymeric ferritin (black arrow heads, **SupplementaryFigure 10A**) and analyzed them using mass spectrometry. Both FTH1 and FTL mirrored the increase in clinically measured ferritin in the matched patients (Figure 6L). We found that native serum ferritin protein in COVID-19 patients was composed of approximately 75.4 ± 10.35% FTL and 24.6 ± 10.35% FTH1 (**SupplementaryFigure 10B**), and that in individuals with ARDS who died had a trend for higher levels of FTL when compared to patients with ARDS who survived (Figure 6M). These data indicate that the elevated serum ferritin in COVID-19 is predominantly a higher ratio of FTL:FTH1 and thus the trajectory of how the molecular composition of serum ferritin changes over the course of disease may have clinical significance and reflect the underlying biological changes we observe in our murine studies (Figure 6N).

## CONCLUSION

In this study, we link macrophage ferritin metabolism and ferroptotic cell death to the regulation of the immune response in ARDS. Using a large COVID-19-associated ARDS data set, we first show that high extracellular ferritin levels in the serum associate with increased mortality in intubated COVID-19 patients with ARDS. These findings are consistent with the findings of others showing that higher serum ferritin levels in patients who developed respiratory failure or died from COVID-19^81, 82^ or non-COVID-19 associated ARDS^43^. Elevated serum ferritin has long been observed in other inflammatory diseases and has largely been overlooked as a non-specific inflammatory marker with unclear effects. In this study, we provide evidence that the regulation and balance of macrophage intracellular and secreted extracellular ferritin directly modulates lung inflammation and injury development in ARDS.

We first show that ferritin transcripts are highly enriched in monocytes and macrophages in the lung and peripheral blood of patients with critical COVID-19 and that this enrichment associates with COVID-19 severity. Macrophage and neutrophil iron loading^23^ (which leads to increased intracellular ferritin) increases injury in experimental ALI models, but to our knowledge, macrophage ferritin expression and modulation has yet to be examined in COVID-19 or ARDS. To mechanistically interrogate the role of macrophage ferritin in the pathobiology of ARDS *in vivo*, we utilized a HALI model to show that hyperoxia exposure increases lung macrophage ferritin expression which correlates with a rise in serum ex-ferritin in similar manner to human ARDS. We focus our efforts on ferritin heavy chain or FTH1 as this particular subunit was the most highly regulated in human and murine macrophages and is essential for survival. Critically, mice with a loss of FTH1 in myeloid cells (*Fth1*^*ΔLysM*^ mice) or resident lung (*Fth1*^*Δcd11c*^) macrophages have improved survival in hyperoxia. This is in line with previous studies that have shown that *Fth1*^*ΔLysM*^ mice are protected from mortality in a cecal ligation-and-puncture (CLP) model of sepsis^77^ as well as from diabetes in a high fat diet-induced obesity model^83^. Both of these prior studies showed dampened inflammatory activation as represented by macrophage cytokine expression and systemic cytokine levels. In this study, we also show that *Fth1*^*ΔLysM*^ mice have decreased lung injury severity outcomes as well as lower inflammatory markers and cytokine levels in the lung in response to hyperoxia.

We present two possible hypotheses for the mechanism of this protection. We first show that FTH1-deficient macrophages resist ferroptotic cell death, which in turn alters macrophage-neutrophil interactions in the injured lung. Prior studies have implicated lung macrophage death, including pyroptosis^84^ and necroptosis^85^ in mediating acute lung injury and ARDS, including COVID-19 ARDS^86^, amplifying lung inflammation through the generation of mediators and damage-associated molecular patterns (DAMPs) in a vicious cycle. Ferroptosis is another form of regulated programmed cell death and drugs or molecules that inhibit ferroptosis alleviate experimental lung injury *in vitro* and i*n vivo*^87, 88, 89^. In some studies, ferritin is required for protection against ferroptosis^90^. In other studies, ferritin increases with ferroptosis inducing agents^67^ and ferritin breakdown by ferritinophagy activates ferroptosis^91^. Iron-rich ex-ferritin can also be secreted in exosomes in response to ferroptotic stress^34, 92^ or excess iron challenge^93^ to reduce intracellular iron levels, thereby helping cells become more resistant to ferroptosis. Few of the above studies focus on immune cells or distinguish between FTH1 and FTL and address if iron availability plays a direct role. To the best of our knowledge, this is the first study to deplete ferritin and impair ferritin breakdown via NCOA4 specifically in macrophages *in vivo* and in the setting of ARDS. *In vitro* studies suggest that macrophage iron loading increases hyperoxia-induced cell death and hyperoxia impairs the capacity of macrophages to sequester iron in ferritin *in vitro*, supporting a role for iron availability in the response of macrophages to hyperoxia^22^. Our findings that a loss of FTH1 increases resistance to macrophage ferroptosis *in vivo* and that systemic iron chelation and inhibiting ferritinophagy exacerbates injury in response to hyperoxia suggest that FTH1 promotes macrophage ferroptosis *in vivo* and that this may not be related to the regulation of intracellular iron levels. Others have also shown that ferroptotic death of macrophages and neutrophils worsens disease in models such as sepsis, cancer, and infection^94^. Consistently, we show that mice with a loss of macrophage FTH1 have a dampened lung cytokine and cellular response to hyperoxia suggesting macrophage FTH1-mediated ferroptosis worsens ALI. Our results also support prior observations that NCOA4 is activated by hyperoxia^95^ as a potential protective mechanism and may mediate ferritin secretion under iron-rich conditions^93^.

The second potential mechanism of protection of the *Fth1*^*ΔLysM*^ mice to hyperoxia involves macrophage ferritin secretion. This secretion which may vary in the composition of FTH1:FTL may initially be a protective mechanism to alleviate intracellular iron overload and escape ferroptosis^33^. However, in the context of the injured lung, the increased presence of iron rich ex-ferritin may in turn have detrimental downstream effects. Prior studies have shown that ex-ferritin (isolated from equine spleen) can trigger neutrophil extracellular trap-mediated cytokine storm in adult-onset Still’s disease^96^ and in sepsis-associated ALI and that ferritin-associated iron induces neutrophil dysfunction in patients with iron overload^97^. Others have shown that ferritin primes inflammasome activation in macrophages *in vitro*, and inflammasome activation results in increased ex-ferritin release^43^. Similarly locally organized and activated FTH1^high^ neutrophils aggravate lung injury in an IL-10-dependent manner^23^ supporting our hypothesis that FTH1 may regulate neutrophil inflammation in ALI. The above studies do not directly link macrophages as the source of ex-ferritin, do not distinguish between FTH or FTL or the iron content of this ex-ferritin, and do not speculate what the receptors for ex-ferritin are. Our findings suggest that the balance between FTL and FTH1 ex-ferritin and the iron content of this ex-ferritin is important for the downstream signaling pathways induced as a result of ex-ferritin secretion by macrophages. Further studies are needed to assess if the iron content of ex-ferritin in individuals with ARDS or if macrophage-secreted FTH1 is consumed by neutrophils in the injured lung which in turn promotes a more injurious neutrophil profile.

Our finding that macrophages secrete FTL in response to a loss in FTH1 is an interesting finding that has been observed previously in *Fth1*^*ΔLysM*^ mice^77^ and may have important consequences for developing therapeutic agents that target ferroptosis. Ferritin does not contain a traditional signal reception particle motif and therefore is not secreted through the endoplasmic reticulum-Golgi apparatus system; but instead, ferritin is secreted in nonclassical pathways via the multivesicular body-exosome system^32, 44^ by macrophages. NCOA4-mediated processing has also been shown to regulate ferritin secretion by extracellular vesicles^93^. Multiple receptors for FTH1 have been discovered in various cell types^98, 99^, supporting the hypothesis that ferritin may not just be an intracellular iron storage complex and can be secreted intentionally. Although receptors for FTL have yet to be described, FTL is protective against ferroptosis in some disease models^75, 76^ and has anti-inflammatory properties^77^. Our results suggest a similar trend, with decreased extracellular iron levels in our myeloid FTH1-deficient mice a plausible explanation for the protective role of FTL in ferroptosis by sequestering available extracellular iron, and a rationale which is consistent with the macrophage’s duty as a regulator of tissue iron homeostasis. Consistently, transplantation of FTL-enriched BALF into wildtype mice mitigated some of the effects of hyperoxia suggesting a possible therapeutic benefit.

Marked elevated serum ferritin, or hyperferritinemia, is observed in several other human diseases, but the structural composition of the extracellular ferritin polymer in COVID-19 and other hyperferritinemic diseases remains to be determined. Our study represents the first study to characterize extracellular ferritin in COVID-19-associated hyperferritinemia. Routine clinical laboratory measurements of “ferritin” are typically ELISA-based on and are not specific for either FTH1 or FTL. We show for the first time that while serum ferritin in COVID-19 contained both FTH1 and FTL, it is predominantly composed of FTL. This supports our hypothesis that FTL may play a role in the regulation of ferroptosis of macrophages and perhaps other cells in ALI. Notably, this hypothesis does not necessarily contradict the observation that higher serum ex-ferritin, and thus higher serum FTL, associating with worse outcomes in COVID-19 ARDS, as this FTL upregulation may represent a failed adaptive or rescue strategy for more severe disease.

There are some limitations to this study. Our primary COVID-19 cohort was built in a single US city, and while it includes subjects from both a quaternary academic center and a community hospital, the study population may not be representative of patients in other care settings, especially in rural and resource-limited settings. Our cohort was also built early in the pandemic (March through May 2020), when optimal care algorithms have yet to be developed for severe COVID-19 infections. We performed bulk RNA-Seq on BMDMs following 3-days of M-CSF treatment. 3-days is a relatively short duration of M-CSF-driven differentiation from myeloid precursors and was chosen because we wanted to maintain the “training” effect of hyperoxia on myeloid progenitors^100^ as much as possible. Studies suggest that at as early as 3 days monocyte markers such as CD14, the human correlate to CCR2, can be identified on BMDMs treated with M-CSF^101^, but whether these cells are representative of monocytes or monocyte-derived macrophages in our model is unclear. Nevertheless, we found significant differences between BMDMs generated from mice in hyperoxia and those from room air mice, as well as similar transcriptional changes induced by hyperoxia between BMDMs and BAL cells, demonstrating that there is some retention of the exposure effect despite the *ex vivo* nature of the study.

In conclusion, in this study we propose that upregulation of macrophage ferritin and secretion of ex-ferritin by macrophages may serve as a crucial immunomodulatory mechanism by which macrophages protect themselves from ferroptosis, regulating neutrophil inflammation and thereby ensuring a regulated immune response to organ injury. Additional work is needed to examine the modulation of ferritin expression and secretion as a therapy for ARDS and other similar diseases.

## METHODS

### New York Presbyterian COVID-19 cohort

In the retrospective component of this study, we identified patients who had confirmation of Sars-Cov-2 infection by reverse transcriptase polymerase chain reaction assays performed on nasopharyngeal swabs. Patients included in our cohort were 18 years of age or older and had an emergency room visit or hospitalization with admission dates between March 3, 2020, and May 15, 2020, at two hospitals in New York City: New York Presbyterian-Weill Cornell Medicine, an academic quaternary care center, and New York Presbyterian-Lower Manhattan Hospital, an academic community hospital. For patients with multiple admissions during this period, only data from the first admission were used. Patients with “do-not-intubate” orders were excluded. The Weill Cornell Medicine (WCM) Institutional Review Board (IRB) approved this study and waived the requirement for informed consent for this portion of the study because the study was minimal risk and could not have been completed practically without a waiver.

#### Data collection.

Relevant data were manually abstracted from the electronic health record by trained research personnel using a quality-controlled protocol and structured abstraction tool^102^, comprising demographic data, clinical characteristics, vital signs, comorbidities, laboratory measurements, and other relevant patient data. Additional data were collected from the Weill Cornell-Critical Care Database for Advanced Research (WC-CEDAR) and the Weill Cornell Medicine COVID Institutional Data Repository (COVID-IDR)^103, 104^.

#### Statistical analysis.

Patient clinical characteristics were compared across strata defined by ferritin level, number of ferritin measurements, and clinical outcome. Patient strata were compared using Wilcoxon rank sum tests or Kruskal-Wallis rank sum tests for continuous variables, Pearson’s Chi-squared tests for categorical variables with all expected cell counts ≥ 5, and Fisher’s exact tests for categorical variables with one or more expected cell counts < 5. Significance values for Fisher’s exact tests were simulated using Monte Carlo simulation when appropriate. The significance threshold for all null hypothesis tests was set at 0.05.

We compared patient- and cohort-level trajectories of ferritin measurements to progression of clinical characteristics during hospital stay. Patient ferritin trajectories were plotted on the log_10_ scale and smoothed using nonparametric penalized cubic regression splines, implemented with the R package *mgcv*^105^. When patients were stratified by clinical characteristic, smoothing was performed within defined strata. In visualizations, trajectories were truncated at 80 days post-admission due to sparsity of ferritin data beyond that point. We also derived three ferritin measurements specific to our cohort to facilitate comparison of ferritin levels across patients. Baseline ferritin was defined as a patient’s first ferritin measurement within 21 days of admission; maximum ferritin was defined as a patient’s highest ferritin measurement during their entire admission; and delta ferritin was defined as the difference between a patient’s first and last ferritin measurements within 21 days of admission. Distributions of derived ferritin measurements were compared across patient strata using violin plots, with stratum-specific medians indicated and compared using Kruskal-Wallis rank sum tests. All analyses were performed using R version 4.0.2^106^. Plots were created using the R package *ggplot2*^107^, and tables were constructed using the R package *gtsummary*^108^.

### Single-cell RNA-Seq analysis

#### Lung macrophages.

Single-nucleus RNA-seq data from lung tissues of 19 COVID-19 decedents and 7 control patients were retrieved from the Gene Expression Omnibus (GEO) under accession number GSE171524^47^. Gene-UMI count matrices from individual samples were merged into a single dataset and processed using the Seurat package. Cells with fewer than 300 detected genes and genes expressed in fewer than 50 cells were excluded to remove low-quality data. Data normalization, variable gene identification, and cell-cycle phase regression were performed using the SCTransform function. To correct for batch effects, the Harmony algorithm^109^ was employed. Dimensionality reduction was achieved using principal component analysis (PCA), and the top 11 principal components (PCs) were utilized to construct a nearest-neighbors graph. Clustering was performed using the Louvain algorithm with a resolution parameter set to 0.8, allowing for the identification of distinct cell clusters.

#### Peripheral blood mononuclear cells.

Processed single cell RNAseq data^46^ were downloaded from Array Express under accession number E-MTAB-10026 and subsequently loaded into Seurat version 3.2.2. Feature plots of *FTH1* and *FTL* expression across all cell types were made on UMAP embeddings using Seurat “FeaturePlot” function and subsequently the CD14+ Monocyte cell population across Status (both Healthy and Covid-19) was selected from the entire Seurat object. Violin plots of FTH1 and FTL expression across Status and Patient Status on Day of Collection were made using Seurat function VlnPlot and statistics were calculated using pairwise wilcox tests with fdr correction applied.

### Experimental animals and models

#### Animals.

*Fth1* floxed (*Fth1*^*fl/flI*^) mice on a C57BL/6J background were kindly provided by the laboratory of Dr. Anupam Agarwal at the University of Alabama at Birmingham (originally generated by Dr. Lukas C. Kuhn, EPFL, Switzerland). They were crossed with *Lysz2*^*Cre*^ mice (also known as LysM-Cre, The Jackson Laboratory, Stock No. 004781) and *Itgax*^*Cre*^ (also known as Cd11c-Cre, The Jackson Laboratory, Stock No. 008068) to generate constitutive *Fth1*^*ΔLysM*^ and *Fth1*^*ΔCd11c*^ mice. *Ncoa4* floxed mice (created and shared by Dr. Joseph Mancias, now deposited in the Jackson Laboratory Stock No. 033295) were crossed with Cd11c-Cre mice to generate constitutive *Ncoa4*^*ΔCd11c*^ mice. In independent experiments, 7–10 week-old sex-matched wildtype C57BL/6 mice (The Jackson Laboratory, Stock No. 000664) with and without treatment with water with 1 mg/mL deferiprone (Chiesi USA) added, *Fth1* knockout mice (*Fth1*^*ΔLysM*^ and *Fth1*^*ΔCd11c*^) and their respective *Fth1*^*fl/fl*^ littermates, and *Ncoa4* knockout mice and their *Ncoa4*^*fl/fl*^ littermates were placed in a custom-build plexiglass chamber, with normal access to food and water, and exposed to hyperoxia using high-flow oxygen, achieving an oxygen concentration > 95% in the chamber. In the bronchoalveolar lavage fluid (BALF) transplant experiments, bronchoalveolar lavage was performed was performed by slowly washing the airways of *Fth1*^*fl/fl*^ and *Fth1*^*ΔLysM*^ mice with PBS. BALF was then centrifuged to remove lavage cells, and 50 μL of the supernatant fluid was intratracheally instilled into anesthetized wildtype mice. Following an hour of observation for recovery, BALF-transplanted mice were exposed to hyperoxia. All murine experiments were approved by the WCM Institutional Animal Care and Use Committee.

### Animal sample collection and analysis

Serum and plasma samples were collected following euthanasia via inferior vena caval access. Bronchoalveolar lavage (BALF) was collected as above and BALF supernatant was analyzed for total protein (Pierce BCA Protein Assay Kit, ThermoFisher 23225), IgM (ThermoFisher 88-50470-88), ferritin (Abcam ab157713), lactate dehydrogenase (LDH, Abcam ab102526), MCP-1 (ThermoFisher88-7391-22), MCP-3 (Abcam ab205571), and MCP-5 (Thermofisher EMCCL12), by ELISA. Serum ferritin was similarly measured by ELISA. BALF pellet was resuspended, the total concentration of cells counted manually, and then subsequently a cytospin preparation was performed to allow differential counting of immune cells. BAL cell death was assayed using a TUNEL assay kit (Abcam ab206386) on cytospin slides. BAL cell 4-hydroxynonenal (4-HNE) immunocytochemistry was performed on cytospin slides (Abcam ab46545).

For histology, lungs were slow inflated with 4% paraformaldehyde (PFA) in PBS and maintained for 15 minutes, following which lungs were carefully extracted from the thoracic cavity. Fixed lungs were embedded in paraffin and sectioned in 5-μm-thick slices. Sections were dewaxed and rehydrated by incubation with xylene and descending ethanol concentrations and then stained with haematoxylin–eosin for histological analysis. H&E-stained lung sections were evaluated and scored by a board-certified veterinary pathologist (S.E.C.) blinded to the genotype and treatment group using a semiquantitative histopathology scoring system used for mouse models of ARDS and SARS-CoV-2^54, 110^. Briefly, six random fields of the lung lobe at 200× to 400× total magnification were chosen and scored for histopathological changes. Bronchiolar epithelial necrosis was assigned using the following tiers: 0, within expected limits; 1, uncommon, <5%; 2, detectable in 5–33%; 3, detectable in 34–66%; and 4, detectable in >66% of lung fields^54^ Lungs were graded for the presence of proteinaceous debris and fibrin, hyaline membranes, neutrophils in alveolar and interstitial spaces, alveolar epithelial necrosis, and macrophages in alveolar and perivascular/peribronchiolar spaces using histopathological scoring system for acute lung injury and diffuse alveolar damage^110^, with final scores obtained by averaging six fields per mouse. An Olympus BX45 light microscope was used to capture images with a DP26 camera using cellSens Dimension software (v1.16).

### Iron quantification

Samples are prepared by adding a digestion buffer, 50% nitric acid by volume with 0.1% digitonin (Sigma-Aldrich D141) to the sample in a 1:1 ratio, then heated at 70°C for 2 hours in a heat block. The digested samples are then removed, cooled to room temperature, and centrifuged for 5 minutes at 6000 xg. The supernatant is collected and then diluted in dilute 0.2% nitric acid for measurement via the graphite furnace atomic absorption spectrometer (PerkinElmer model 900z). Iron is measured in the digestion buffer to assess iron contamination, and sample iron levels are quantified using a standard curve of known iron concentrations.

### RNA extraction and real-time polymerase chain reaction

Total RNA was extracted from BMDMs or AMs using Qiagen RNeasy Micro Kit and reverse transcribed into cDNA using a High Capacity cDNA Reverse Transcription kit (Life Technologies). Real-time PCR was performed using qPCR master mix (Life Technologies) on a ABC instrument with TaqMan gene expression primers for *Fth1* (Mm00850707_g1) and *Ftl* (Mm03030144_g1).

### Western blots and antibodies

Cell lysates were generated from BMDMs and BAL cells from room air and mice exposed to hyperoxia via sonication and RIPA Cell Lysis Buffer (ThermoFisher 89901). These lysates were then boiled at 100°C for 5 minutes in loading buffer containing SDS and following resolution on 4–12% SDS-PAGE gels, transferred onto nitrocellulose membranes. Specific proteins blotted for include FTH1 (CST 3998), FTL (Abcam 109373), TFRC (Abcam 214039), SLC7A11 (CST 12691), GPX4 (Abcam 125066), and BACTIN (Sigma Aldrich A2228).

### Bone marrow-derived macrophage experiments

Bone marrow cells were isolated from the murine femur and tibia through a 25 G needle with a 12 ml syringe filled with complete DMEM attached (Life Technologies). Cell aggregates were removed using an 18 G needle and 70 μM cell strainer, after which the single cell suspension was centrifuged at 1200 rpm for 5 min. Cells were plated in complete DMEM supplemented with 10 ng/mL M-CSF for 7 days for *in vitro* experiments, for 3 days when followed by RNA isolation and RNA-Seq analysis.

#### In vitro experiments.

BMDMs were treated with 1 μM RSL3 (Selleckchem, Catalog No.S8155) for 4 hours. Viability was assessed using the AlamarBlue Cell Viability Reagent (ThermoFisher DAL1025) or PrestoBlue Cell Viability Reagent (ThermoFisher A13262) where indicated according to manufacturer protocol. *In vitro* hyperoxia was modeled using a Stemcell Hypoxia Chamber (Catalog# 27310) and oxygen with 5% CO_2_ mixed in, filled until achieving a chamber oxygen concentration of greater than 90%. The cells are then incubated for 24 hours, with the oxygen sensor left in to ensure that oxygen levels do not fall during the incubation.

#### Bulk RNA-Seq analysis.

Sample files were checked for sequence quality using FastQC^111^. The resulting reads were mapped to the mouse reference genome GRCm38 using STAR^112^ aligner and gene-wise expression counts generated using the “-quantMode GeneCounts” parameter. After filtering for lowly expressed genes, the R package edgeR^113^ was used to perform differential gene expression comparisons and calculate FPKM values normalized by library size. Significance was defined by adjusted p-value (false discovery rate or FDR) < 0.05 and fold change > 1.25. Differentially expressed genes were illustrated via Volcano plot using VolcaNoseR^114^. Gene ontology (GO) and Kyoto Encyclopedia of Genes and Genomes (KEGG) analysis was performed using ShinyGO^115^.

### Ferritin isolation and characterization

For ferritin isolation, serum was buffered by adding 0.05M sodium acetate in a 1-to-1 ratio, and then adding 1M acetic acid to reach a target pH of 5. This buffered serum was subsequently heated at 70 °C for 10 minutes (9), and then centrifuged at 15,000 xg for 30 minutes. The resultant pellet of denatured protein was discarded, and protein from the supernatant was precipitated using cold acetone. Acetone at −20 °C was added to the heated serum supernatant at a 4-to-1 ratio by volume, and this mixture was incubated at −20 °C for one hour. This was then centrifuged at 15,000g for 10 minutes, and the supernatant acetone was carefully removed by pipetting. The protein pellet was left to air dry for no more than 15 minutes to allow the residual acetone to evaporate, and then resuspended for native gel electrophoresis. Bands excised following standard Coomassie Blue staining protocol^116^ were submitted for mass spectrometry analysis (*see Supplemental Methods)*.

## Supplementary Material

This is a list of supplementary files associated with this preprint. Click to download.

- SupFiguresCompositewithLegends10.15.24compressed.pdf

## Figures and Tables

**Table 1.** Clinical outcomes by delta ferritin in ICU patients.

*Parameter/Ferritin (ng/mL)	*Above median delta ferritin (n=182)	*Below median delta ferritin (n=182)	**p-value
Delta ferritin	+398 (85, 921)	−512 (980, −220)	< 0.001
28-day Morality n (%)	67 (37%)	33 (18%)	<0.001
Days Free from Ventilator	0 (0, 9)	0 (0, 14)	0.019
P/F Ratio	121 (86, 174)	112 (86, 167)	0.4
Driving Pressure	15 (12, 18)	14 (11, 17)	0.3
SOFA (day on intubation)	11 (10, 13)	11 (11, 13)	0.2
SOFA (72hr)	11 (10, 13)	11 (9, 12)	0.2
Renal Replacement Therapy n (%)	49 (27%)	38 (21%)	0.2
Venous Thromboembolism n (%)	44 (24%)	39 (21%)	0.5

* Statistics presented: N (%); median (IQI)

** Tests used: Wilcoxon rank sum test; Pearson’s Chi-squared test.

SOFA=sequential organ failure assessment; PF=PaO_2_/FiO_2_
